# Possible Mechanisms by which Stefin B could Regulate Proteostasis and Oxidative Stress

**DOI:** 10.3390/cells8010070

**Published:** 2019-01-18

**Authors:** Eva Žerovnik

**Affiliations:** Department of Biochemistry and Molecular and Structural Biology, Jožef Stefan Institute, Jamova 39, 1000 Ljubljana, Slovenia; eva.zerovnik@ijs.si; Tel.: +386-1-477-3753

**Keywords:** cystatin B, amyloid, oligomers toxicity, protein aggregation, autophagy, oxidative stress

## Abstract

Human stefin B is a protease inhibitor from the family of cystatins. It was reported that it forms oligomers in cells. We have shown that it has a role in cell’s response to misfolded proteins. We also have shown that its oligomers bind amyloid-beta (Aβ). Here, we discuss ways, how stefin B could reduce build-up of protein aggregates by other proteins and consequently reduces ROS and, how this might be connected to autophagy. When overexpressed, stefin B forms protein aggregates itself and these protein aggregates induce autophagy. Similarly, cystatin C was shown to bind Aβ and to induce autophagy. It is also suggested how more knowledge about the role of stefin B in a cell’s response to misfolded proteins could be used to modulate progressive myoclonus epilepsy of type 1 EPM1 disease.

## 1. Introduction

Human stefin B is a protease inhibitor, belonging to the family of cystatins. Human cystatins are of three types: cystatins of type 1 and 2 and kininogens. By the MEROPS classification [1] subfamily I25A designates intracellular cystatins A and B and subfamily I25B, secreted cystatins C. Human cystatin C (cysC) is a known amyloidogenic protein. Its mutant L68Q causes hereditary cystatin C amyloid angiopathy (HCCAA), also called hereditary cerebral hemorrhage with amyloidosis, Icelandic type. Its role in Alzheimer’s disease (AD) was reviewed by Kaur & Levy [2]. The possible ways in which cysC could play a protective role was discussed: either it would act by inhibition of cysteine proteases, inhibit Aβ oligomerization and amyloid-fibril formation, or it would induce autophagy. As for this latter possibility, using multiple methods, it was demonstrated that cysC induces autophagy in cells under basal conditions, and enhances the autophagy in cells exposed to nutritional deprivation and oxidative stress [3,4].

Human stefin B (cystatin B) mutations cause progressive myoclonus epilepsy of type 1 (EPM1) [5,6]. While most mutations in the cystatin B (*CSTB*) gene are dodecamer repeats [7], which contribute to lower translation of the protein and lack of function, other kind of mutations were also observed: frame-shift, missense and splice mutants [8]. In the case that at least one allele codes for the missense mutant, this can become expressed up to 40% of the usual expression level [8]. Thus, due to the propensity to aggregate of some EPM1 mutants (R68X, G50E, G4R, Q71P) (Table 1) protein aggregation may contribute to the disease phenotype [9].

In our hands, human stefin B was used extensively as a model amyloidogenic protein for folding and protein aggregation studies [10,11,12,13,14,15,16]. However, aggregation of stefin B and some EPM1 mutants in different cell lines was also studied [9,17,18]. It was found that the gain in toxic function applies [18].

In this short review, we explore how stefin B could regulate proteostasis and oxidative stress. It was reported that stefin-B knock-out KO cells are more sensitive to oxidative stress [19] and we have found ourselves that mural stefin B KO astrocytes bear more protein aggregates (by other proteins) and have impaired autophagy [20]. In Section 2 and Section 3, we talk about the cause and pathology of EPM1 (Unverricht–Lundborg) disease, because stefin B (*CSTB* gene) was found mutated in some EPM1 patients and is one of two known genes causing this disease. In Section 3 and Section 4, we present our in vitro studies on amyloid fibril formation by the wild-type stefin B and some of the EPM1 mutants, which does not necessarily mean this has anything to do with the disease. The fact that this protein aggregates, as many other proteins do, can be used mainly as a model for amyloidogenic proteins. However, stefin B expression in cells and properties of the intracellular missense EPM1 mutants aggregates are relevant and represent possible modulating factors of the course of the disease (gain in toxic function). Indeed, most mutations lead to lower protein expression and thus loss of protease inhibitory function [21]. Nevertheless, even if loss of function would be the main cause of EPM1 symptoms, it is very important to understand the putative alternative function of stefin B’s oligomers.

## 2. Progressive Myoclonus Epilepsy of Type 1 (EPM1)—Unverricht–Lundborg Disease

Progressive myoclonic epilepsy of type 1 (EPM1), also known under the name of Unverricht–Lundborg disease, is a type of progressive myoclonus epilepsy (PME). EPM1 is inherited in autosomal recessive manner and exhibits signs of myoclonus, epilepsy, and progressive neurodegeneration. Pathological examination in patients has shown a marked neural loss of Purkinje cells in the granular layer of the cerebellum [22]. Patients experience jerks, tonic-clonic seizures, and progressive cognitive decline. Depressive moods are also frequently observed. The disease is spread mainly in the Baltics and Mediterranean. Symptoms appear as early as 12 and up to 18 years of age. The disease can lead to dysarthria, and ataxia in later stages [23].

The cystatin B (*CSTB*) gene is located in chromosome 21q22.3. At least 10 mutations in the *CSTB* gene have been reported, as observed in some EPM1 patients [8]. The most common change is the dodecamer repeat expansion in the promoter region of the gene, which finally leads to reduced protein levels. Other kind of mutations of stefin B are shown in Table 1, such as missense, non-sense, frame-shift/truncation and spliced.

## 3. Disease Development and Progression

It is not always clear whether the mutated stefin B protein is responsible for the disease development and progression by exerting toxicity by its protein aggregates or, lack of the protease inhibitory function or, a combination of both. It is important to clarify protein’s alternative function(s), such as its role in the multiprotein complex involved in regulation of the cytoskeleton [24], anti-oxidative stress effect [19] and putative chaperone-like function [25,26]. Stefin B was shown to bind to histones and to indirectly regulate the cell cycle through inhibition of cathepsin L in the nucleus [27]. If such functions would be omitted in EPM1 patients, the consequences could lead to neurodegeneration.

Stefin B deficient mice (knock-out—KO mice) serve as a reasonable model for EPM1 disease, KO mice develop myoclonus after 1 month and ataxia after 6 months. Myoclonus occurs only during sleep with no tonic-clonic seizures, as well as no photosensitivity nor spike waves in the electroencephalograph (EEG) [28]. Stefin B deficiency triggers several secondary processes, such as overstimulation of serotoninergic transmission [29] and a defect in dopamine transmission [30], which may lead to the seizure phenotype of EPM1. Of note, the hippocampal slices from *CSTB*-deficient mice are hyperexcitable, displaying pronounced epileptic spike and waves patterns. As well, GABA-nergic inhibitory neurons are highly susceptible to seizure-induced neural damage, which may reflect in the progressive nature of this disease [31]. In patients, cerebral atrophy and reduced levels of grey matter in the motor cortex and thalami and reduced cortical thickness have been observed [32]. Interestingly, the disease has been linked to obesity [33]. To our knowledge, stefin B knock-in mice have not yet been developed.

## 4. Amyloidogenesis of Human Stefin B In Vitro

Human stefin B is rather labile and more amyloidogenic in comparison to stefin A, the two members of the family I25 of cystatins. It has served as a good model protein for studies of protein aggregation to amyloid fibrils. Size and morphology of the prefibrillar aggregates are in agreement to other such amyloidogenic proteins [34]. In accordance to other studies, the prefibrillar oligomers of stefin B were found toxic [34,35]. A likely mechanism for the amyloid cytotoxicity is membrane interaction and damage [36]. As observed for a number of amyloid forming proteins, stefin B bound to and perforated acidic phospholipid bilayers [27,37] in vitro.

As described in the Introduction, we have studied several EPM1 mutations of stefin B. In order to study their stability and aggregation propensity, the mutant proteins were expressed in *E. Coli* and purified using affinity and size-exclusion chromatography. By activity measurements it was found that the missense mutant G4R was not active, while G50E and Q71P showed lowered inhibitory activity. The natively unfolded fragment R68X aggregated extensively, however, it was cleared from the cells rather fast [38,39].

By measuring CD, it has been shown that G50E and Q71P mutants possess similar secondary structure but a more open tertiary structure compared to the wild type (likely a pre-molten globule). Our previous studies showed that the R68X fragment was natively unfolded and that it transformed into amyloid fibrils already at neutral pH [40]. Interestingly and in distinction to other examples of the missense EPM1 mutants, G4R was found correctly folded and of similar stability as the wild type but it demonstrated a prolonged (4×) lag phase of the aggregation reaction [40].

## 5. Protein Aggregation in Cells and Oxidative Stress

The ex vivo (cell culture) studies have shown that even the wild type stefin B forms small scattered aggregates when over expressed, which increase in amount upon proteasome inhibition [17]. Compared to the wild type, all the missense EPM1 mutants formed larger cytosolic aggregates. They were frequently perinuclear and reminiscent of aggresomes [17]. In contrast to the belief that aggresomes are cytoprotective [41], in our case the cells with more abundant aggregates were less viable. Partial colocalization of stefin B aggregates with LC3 is in agreement to the notion that the aggresome-like aggregates are targeted for autophagic clearance [42].

Scattered smaller aggregates correlated with folded protein and bigger, aggresome-like perinuclear inclusions formed with intrinsically unfolded protein, such as the fragment R68X or pre-molten globular type conformation, such as Q71P. Of note, the mutants and even the wt, which formed scattered (soluble) aggregates, exerted increase in ROS levels [18].

Why ROS increase when proteins aggregate can be explained by damaged mitochondrial and lysosomal membranes (still remains to be shown) as well as Fenton reaction when Fe^2+^ ions bind to the aggregates and H_2_O_2_ is formed [43,44,45]. In the case of stefin B, we have shown that Cu^2+^ bound to prefibrillar aggregates and inhibited their transition to mature fibrils [46].

It is to be noted that stefin B deficiency also increases ROS. However, this may be due to lack of the protein’s alternative function in protecting cells of other protein’s aggregates [20].

## 6. Protein Aggregation and Autophagy

Our study of KO mice astrocytes showed that in absence of stefin B other proteins form more aggregates [20]. It also was found that autophagy was less efficient and that this could be repaired by adding stefin B active oligomers from monomer to tetramer. The same oligomers from G4R mutant, which is not active as protease inhibitor, did not have much effect to autophagic flux. From these studies [20] we conclude 1st that stefin B antiprotease function is important for autophagy to progress normally and 2nd that stefin B somehow reduces aggregates of other proteins, lowers ROS and increases cell viability, which perhaps can be explained by its alternative functions.

Recently, Chauhan and coworkers [47] reported that under oxidative and proteotoxic stress conditions, TRIM16 activates ubiquitin pathway genes and p62 via NRF2, leading to ubiquitination of misfolded proteins and formation of protein aggregates [47]. Furthermore, they have shown that TRIM16 acts as a scaffold protein, which interacts with p62, ULK1, ATG16L1, and LC3B and in such a manner facilitates autophagic degradation of protein aggregates.

Sherman and coworkers reported that even early aggregate formation on the level of lower oligomers is regulated [48]. These authors have shown that cells under proteotoxic stress activate different pathways, helping aggresome formation and inducing autophagy. It is known that Bag3 recruits Hsp70-bound protein aggregates and links them to dynein motor that drives the microtubular transport of the cargo. They report a novel finding that the complex Hsp70-Bag3 regulates kinase LATS1. It was demonstrated that this system regulates formation of early aggregates before they get transported to the aggresome. The increase of aggregates size was observed by using super-resolution imaging by photoactivation localization microscopy (PALM). Depletion of the kinase LATS1 led to significant increased clusters or the aggregates, while presence of LATS1 repressed early stages of protein aggregation on the level of few monomers [48].

## 7. Is Protein Aggregation a Modulating Factor in Myoclonal Epilepsies and Broader?

Human stefin B in its monomeric form acts as a protease, cysteine cathepsins, inhibitor. Lower oligomers still possess the antiprotease activity and it has been shown that they repair the autophagic flux, which got disturbed in stefin B KO astrocytes [20]. Concominant with impaired autophagy, more scattered protein aggregates appeared in stefin B KO cells. We propose that the protein aggregates raise oxidative stress, which is observed in stefin B KO cells, KO mice and EPM1 patients bearing stefin B mutations [19]. We also have shown that stefin B oligomers bind Aβ [25], and a function of an amateur chaperone has been proposed for stefin B oligomers [49]. Therefore, lack of stefin B alternative function would lead to more Aβ aggregates and raised ROS. Interestingly, stefin B when over expressed forms protein aggregates itself, however, it at the same time induces autophagy and cell death does not increase much, in comparison to some EPM1 mutants (G4R and G50E), whose aggregates also raise ROS [9,18].

In the two papers by Polajnar & Žerovnik, 2011 and 2014 [50,51], we suggested that proteinopathies may be broader than neurodenerative diseases, including progressive myoclonus epilepsies (PMEs) and neuropsychiatric diseases, such as schizophrenia. A common denominator would be impaired autophagy.

**Scheme 1:**
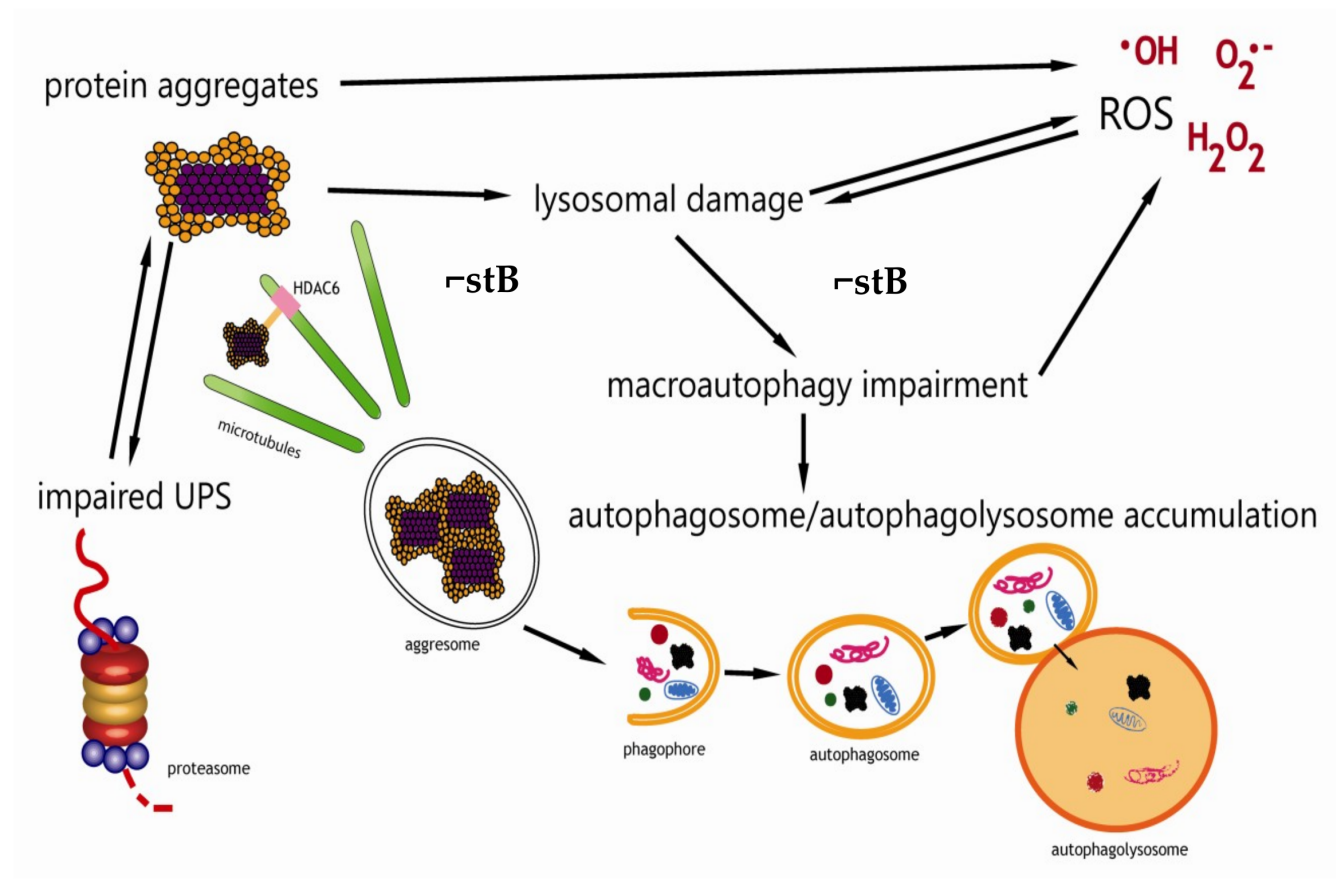
Link between protein aggregation, increased ROS and autophagy. The combined events of oxidative stress and UPS overload signal induction of autophagy. In functional autophagy, lysosomes fuse with autophagosomes to form autolysosomes. Autolysosomes have a low pH that activates various proteases to degrade cell debris accumulated in the autophagosomes. However, due to damaged lysosomal membranes, autophagosomes accumulate because they are unable to form autophagolysosomes. Together with dysfunctional autophagy and impaired UPS, a vicious circle is created. Copyright: Trends in Molecular Medicine; adapted from Polajnar & Žerovnik TMM, doi:10.1016/j.molmed.2011.02.005.

In accordance with our opinion [50,51], autophagy-enhancing substances could be beneficial for most PMEs, as is the case for neurodegenerative diseases [52,53]. One such drug is rapamycin, a well-known inhibitor of mTOR (see Scheme 1), which was reported to alleviate toxicity of a range of aggregating peptides [52]. Unfortunately, it is also associated with severe side effects, such as long-term immunosuppression. One study reported the identification of small molecule compounds that enhance the effect of rapamycin to be used in smaller concentrations [54].

Taken altogether, we suggest to add drugs directed against oxidative stress, metal imbalance and especially to enhance autophagy (Scheme 1), as adjunctive trial therapy for PMEs.

Of note, beneficial effects of heat and antioxidants have been observed with some patients with EPM1 (R. Kalviäinen and I. Ravnik—personal communication). This could argue in favor of the oxidative stress hypothesis [19] as well as proteotoxicity, stemming from protein aggregation. When the stress response is turned on by heat (heat shock proteins, including chaperones) the aggregates would get cleared and oxidative stress diminished.

## Figures and Tables

**Table 1:** Mutations in *CSTB* underlying Unverricht–Lundborg disease (EPM1) [8].

Mutation	Position of Mutation in Gene/Type	Predicted Consequence on Protein
dodecamer repeat expansion	5’ UTR/expansion	reduced CSTB expression
c.10G>C	exon 1/missense	p.G4R
c.67-1G>C	intron 1/splice site	p.delV23_K56
c.149G>A	exon 2/missense	p.G50E
c.168>A	exon 2/splice site	aberrant splicing?
c.169-2A>G	intron 2/splice site	aberrant splicing?
c.168+1_18del	intron 2/deletion	p.delV23_K56 p.V57EfsX28
c.202C>T	exon 3/nonsense	p.R68X
c.218_219delTC	exon 3/deletion	p.L73FSX3
c.212A>C	exon 3/missense	p.Q71P
